# Using large language models to enhance clinically-driven missing data recovery algorithms in electronic health records

**DOI:** 10.1093/jamiaopen/ooag080

**Published:** 2026-06-02

**Authors:** Sarah C Lotspeich, Abbey N Collins, Brian J Wells, Ashish K Khanna, Joseph Rigdon, Lucy D’Agostino McGowan

**Affiliations:** Department of Statistical Sciences, Wake Forest University, Winston-Salem, NC 27109, United States; Department of Psychology, North Carolina State University, Raleigh, NC 27607, United States; Department of Biostatistics and Data Science, Wake Forest University School of Medicine, Winston-Salem, NC 27157, United States; Division of Critical Care Medicine, Department of Anesthesiology, Wake Forest University School of Medicine, Winston-Salem, NC 27157, United States; Outcomes Research Consortium, Houston, TX 77030, United States; Department of Biostatistics and Data Science, Wake Forest University School of Medicine, Winston-Salem, NC 27157, United States; Department of Statistical Sciences, Wake Forest University, Winston-Salem, NC 27109, United States

**Keywords:** chart reviews, computable phenotype, learning health system, missing data, whole-person health

## Abstract

**Objectives:**

Electronic health record (EHR) data are prone to missingness and errors. Previously, we devised an enriched chart review protocol where a “roadmap” of auxiliary diagnoses was used to recover missing values. Still, chart reviews are expensive and time-intensive, limiting the number of patients whose data can be reviewed. Now, we investigate the accuracy and scalability of a roadmap-driven algorithm, based on International Classification of Diseases, 10th revision (ICD-10) codes, to mimic expert chart reviews and recover missing values.

**Materials and Methods:**

In addition to the clinicians’ original roadmap from our previous work, we consider new versions that were iteratively refined using large language models (LLMs) in conjunction with clinical expertise to expand the list of auxiliary diagnoses. Using chart reviews for 100 patients from an extensive EHR, we examine algorithm performance.

**Results:**

Across 100 chart reviewed patients, there were 413 missing values in the EHR data. The expert chart reviews recovered 49 (12%), while the algorithms using LLMs-enhanced roadmaps recommended almost twice as many (83-89, 20%-22%). The final algorithm using clinician-approved LLMs’ additions offered a balance (73, 18%), expanding the original roadmap with LLMs’ suggestions but only when deemed clinically relevant. When applied to a larger study of 1000 patients from the same EHR, the per-patient median number of non-missing values increased from 6 to 7.

**Discussion:**

Clinically-driven algorithms (enhanced by LLMs) can recover missing EHR data with similar accuracy to chart reviews and feasibly be applied to large samples. Extending them to monitor other data quality dimensions is a promising future direction.

## Background and significance

### Learning health systems and electronic health record (EHR) data

Refining healthcare systems is vital to enhancing care delivery and improving patient outcomes. Learning health systems (LHS) use data and analytics to learn from patients’ data and relay that information back to clinicians, fostering cycles of continuous improvement.^1–3^ Enhancing connections between research and practice is critical to LHS,^2^ and this connection depends heavily on electronic health record (EHR) data.^4^^,^^5^

Electronic health record data present a huge opportunity to operationalize computable phenotypes and advance LHS,^6^ but there are challenges. Because they are routinely collected in a fast-paced clinical environment, EHR data are predisposed to missingness and errors.^4^^,^^7–11^ Key predictors for the phenotype may be measured for some, but not all. Because their measurement depends on clinical decision-making (e.g., physician ordering a test), these predictors are likely missing not at random.^12^^,^^13^

### Whole-person health scores

Our LHS wants to operationalize the allostatic load index (ALI), which is a computable phenotype for allostasis (the body’s ability to adjust to physiological changes and maintain stability).^14^^,^^15^ The ALI is a modifiable index designed to capture physiological “wear and tear” across multiple body systems due to stress.^14^^,^^16^^,^^17^ It can provide clinicians with an early indication of short- and long-term health risk, as the ALI is associated with outcomes including cardiovascular disease, hypertension, diabetes, obesity, and overall risk of morbidity and mortality.^18–25^

We want to apply the calculation from Seeman et al.,^25^ which computes ALI from 10 biomarkers across 3 body systems (Table 1). However, in EHR data, many of these biomarkers are measured infrequently. While errors in non-missing biomarkers are possible, based on our previous studies in this LHS,^26^ we believe missingness to be a bigger issue. We recently assessed how to navigate data challenges and more accurately measure ALI from EHR data by incorporating expert chart reviews.^26^

**Table 1:** Ten components of the allostatic load index (ALI) were defined by discretizing measurements across 3 body systems at clinically-driven thresholds.

ALI component	Threshold	Search term(s)
Cardiovascular system		
Systolic blood pressure	>140	Hypertension
Diastolic blood pressure	>90	Hypertension
Metabolic system		
Body mass index	>30	Obesity; Morbid obesity;
		Grade I, II, or III obesity
Triglycerides	≥150	Hypertriglyceridemia
Total cholesterol	≥200	Hypercholesterolemia
Inflammation system		
C-reactive protein	≥10	Sepsis; infection; Auto-immune
		inflammatory syndrome
Hemoglobin A1C	≥6.5	Diabetes; Impaired
		glycemic control
Serum albumin	≥3.5	(None given)
Creatinine clearance	<110 (Males)	Renal failure;
	<100 (Females)	Insufficiency; Acute
		kidney injury;
		Chronic renal failure
Homocysteine	>50	Hyperhomocysteinemia;
		Vitamin deficiency

If the component was missing from the extracted electronic health record (EHR) data, chart reviewers searched for auxiliary information in the patient’s medical chart in Epic (the institution’s electronic charting software). If the search term was present, the patient was treated as having an “unhealthy” measurement. This table was adapted from our prior work in Lotspeich et al.^26^

### Expert chart reviews

Expert chart reviews (also called source document verification) are a common approach to gauging the quality of routinely collected data.^26–32^ Typically, teams of skilled reviewers manually compare the analytical dataset (e.g., extracted from the EHR) to clinical source documents (e.g., electronic charts) to identify differences. Since the source documents are closer to the point of care, they are generally considered more accurate than the analytical dataset. With a prespecified protocol in place,^32–34^ chart reviews can take weeks or months to complete, and data are often collected in a standardized electronic format, like Microsoft Excel or REDCap.^35^

Chart reviews uncover valuable information about data quality and opportunities for process improvement. Still, they are resource- and time-intensive, which greatly limits their scalability. Our previous chart reviews required the effort of 4 skilled chart reviewers (clinical research technicians) over 6 months to evaluate a focused list of variables (only ALI components) for a small subsample of 100 patients from a larger study of 1000 routine healthcare users from our EHR.^26^ Adding a novel “roadmap” for missing data recovery, created by clinical experts, we filled in some gaps using auxiliary health information (e.g., related diagnoses) or anchors,^36^ but 900 patients’ data were left totally untouched, and even those who underwent chart review could still have missing data. Herein, we explore how (1) expanding the roadmap, potentially with large language models (LLMs) and (2) applying it algorithmically, instead of requiring humans for implementation, can promote this protocol’s efficacy and efficiency.

### Large-scale algorithms in EHR data

Structured EHR data, like International Classification of Diseases, 10th revision (ICD-10) codes,^37^ are especially valuable for patient phenotyping.^38–49^ Often, phenotype development involves model training and deployment using EHR data and a subset of “gold standard” outcomes (e.g., validated diagnoses) obtained through chart reviews.^48^ The final algorithm might involve statistical modeling, machine learning, or artificial intelligence.

Data cleaning algorithms differentiate between plausible and implausible values in EHR data^50–52^ and detect misspellings in text fields.^52–55^ Some algorithms are variable-specific, since they rely on the clinical context to define plausibility.^56–58^

To handle missing EHR data, imputation (i.e., replacing missing values with informative placeholders), which seeks to preserve the size and statistical power of the full sample, is incredibly common.^8^^,^^59–64^ The imputed values (placeholders) range in complexity from summary statistics to model predictions. However, replacing missing values with poor placeholders can actually hurt rather than help analyses.^65^ To recover missing ALI components, we did not want to rely on relationships between variables in our somewhat sparse EHR dataset. Instead, we leverage clinical insights to impute variables based on a prespecified roadmap.

## Objective

Using ALI and healthcare utilization for demonstration, we compare the accuracy and scalability of a missing data recovery algorithm based on a clinically-driven, LLMs-enhanced roadmap of auxiliary ICD-10 codes against expert chart reviews.

## Materials and methods

### Cohort description

Our study builds on a sample of N=1000 adults 18-65 years old who newly engaged in outpatient primary care at Atrium Health Wake Forest Baptist (AHWFB) between March 2018 and 2020. Located in Winston-Salem, North Carolina, AHWFB is a 900-bedded tertiary care hospital system attached to Wake Forest University School of Medicine (WFUSM). This study was approved by the Institutional Review Board at WFUSM.

Electronic health record data were extracted for all patients, including demographics and measurements for ALI components (Table 1). Some components were missing for almost everyone (Supplemental Figure S1A). Errors in non-missing values were possible (e.g., due to the data extraction algorithm) but very uncommon. On average, patients had 6 of 10 non-missing ALI components in the EHR data (interquartile range [IQR] =[5,7], Supplemental Figure S1B). A subset of n=100 patients underwent chart reviews.^26^

### Expert chart review protocol and findings

We previously developed a novel “enriched” chart review protocol incorporating a clinical expert-derived “roadmap” (Table 1) to ensure the quality and completeness of ALI components in EHR data.^26^ It was designed to address 2 key goals:

- *Validation:* For non-missing components, confirm that the extracted value (in extracted EHR data) matches the patient’s chart.
- *Recovery:* For missing components, locate supplemental diagnoses (in electronic charts) that provide information about the unmeasured values.

These goals aligned with 2 data quality dimensions (concordance and completeness) outlined by Weiskopf and Weng.^66^

Expert chart reviewers (clinical research technicians [CRTs]) followed this protocol to manually search patients’ electronic charts. The subsample of 100 patients reviewed were chosen via a multiwave adaptive sampling strategy.^26^ The first 52 were randomly sampled based on whether they engaged in the healthcare system (yes/no) and discretized unvalidated ALI (above/below the median) via a balanced case-control design. The last 48 chosen were those with the largest/smallest residuals based on the naive model (i.e., using unvalidated EHR data only). While selection of these patients was enriched for that particular clinical question, their data should be representative of the original sample with respect to missingness in the ALI components and how much could be recovered, which is of interest here.

In total, 4 CRTs reviewed 11 472 data points (1767 labs and 9705 vitals). These data are “alloyed” gold standards (i.e., presumed to be more accurate than extracted EHR but still imperfect). Non-missing ALI components almost always matched the EHR, and some missing ones were recovered using the roadmap. Still, chart reviews required 100 hours of human effort. Also, while we now feel confident in the non-missing EHR data, missingness remains a key challenge (especially for patients without chart reviews).

### Missing data recovery algorithms

Since completing the chart reviews, we have been pursuing scalable ways to recover missing EHR data across our healthcare system. The clinicians’ search terms from the roadmap in our previous work (Table 1) could be captured by ICD-10 codes.^37^ Therefore, we were interested in developing an algorithm to recover missing ALI components from all 1000 patients based on ICD-10 codes. Computationally, this task is fairly simple: merge the roadmap into patient diagnoses and evaluate potential matches.

Our merging algorithm required all terms to appear somewhere in the ICD-10 code’s description to be considered a match. For example, “vitamin deficiency” required both “vitamin” and “deficiency” to be present. The 20 search terms in the *clinicians*’ *original roadmap* matched 1234 ICD-10 codes, of which 211 were present in our sample (Table 2).

**Table 2:** Comparison of the pros and cons for various data sources, including the extracted electronic health record (EHR) data, expert chart reviews, and various missing data recovery algorithms (by roadmap).

Data source	Addresses errors	Addresses missingness	Sample size	List of diagnoses	Clinical relevance	Statistically efficient	Search terms	Matched in sample (overall)
Extracted EHR data	No	No	Large	Short	High	Maybe	−	−
Expert chart reviews	Yes	Yes	Small	Short	High	No	−	−
Algorithm w/ clinicians’ original	No	Yes	Large	Short	High	Yes	20	211 (1234)
Algorithm w/ LLMs (baseline)	No	Yes	Large	Long	Low	Yes	393	782 (12 151)
Algorithm w/ LLMs (context)	No	Yes	Large	Long	Low	Yes	277	514 (5661)
Algorithm w/ LLMs (context + clinicians)	No	Yes	Large	Long	High	Yes	115	421 (421)^a^

For each roadmap, "Search terms" is the number of proposed search terms, and "Matched in sample (overall)" is the count of International Classification of Disease, 10th revision (ICD-10) codes matching those search terms for patients in our sample (versus in general).

a Clinicians were only asked to review ICD-10 codes from LLMs (context) that matched patients in our sample, so it was not possible for LLMs (context + clinicians) to match more than 514 ICD-10 codes.

This algorithm attempts to recover missing data by replicating the process performed by human chart reviewers. Once established, it is faster, more affordable, and efficiently applies across the entire study. Since costs are not tied to sample size, this algorithm makes large-scale EHR data recovery possible in a way that chart reviews cannot.

However, the roadmap was initially created for use by human chart reviewers with clinical expertise. Therefore, some search terms were expected to be used at the chart reviewers’ discretion based on additional context. For example, one of the search terms for missing C-reactive protein (CRP) was “infection,” which matched ICD-10 codes ranging in severity from systemic (like sepsis) to acute (like upper respiratory infections).

### Enhancements with large language models (LLMs)

Building on this algorithmic foundation, we employed LLMs to expand the list of search terms. For accessibility, we implemented the *LLMs-based roadmap enhancements* in open-source software. Specifically, we used the ellmer package in R, which enables programmatic interaction with LLMs through “tool-calling” and allows for seamless integration with downstream data management and analysis.^67^ As of March 23, 2026, Google was the only provider in ellmer with an unpaid Application Programming Interface (API) tier and their default model was Gemini-2.5-Flash.^68^ Using the Gemini API’s default parameters (temperature =1, top-P =0.95, top-K =64, and candidate count =1),^69^ we tested 2 LLMs enhancements. See Supplementary Material S1 for all R code.

### LLMs (baseline) roadmap without context

First, we prompted the LLMs to generate relevant terms for each ALI component based only on the name of each biomarker and general guidance on whether high or lower values were considered unhealthy (Prompt 1).

Prompt 1: Generate LLMs (baseline) roadmap Please propose an exhaustive list of terms (avoiding acronyms) that will be used to search ICD-10 descriptions to identify each of the missing biomarkers. Create a new data frame with these codes named “df_nocontext_[number].” Be sure to make as exhaustive a list as possible.

As a form of self-consistency,^70^ Prompt 1 was placed inside of a loop and provided to 20 newly initiated chats, allowing the LLMs to independently respond many times. (The daily query limit for the API was 20.) This was preferable over providing a single prompt that asked for 20 new data frames, since previous versions would still be within the context window and could skew or “distract” later ones.^71^ Across the loop, the number of unique ICD-10 codes matching the proposed search terms (taken as the superset of all those proposed thus far) seemed to plateau after about 10 iterations (Supplemental Figure S2).

Ultimately, the *LLMs (baseline) roadmap* took the superset of these 20 dataframes (i.e., the collection of terms that appeared at least once across the 20 times that the LLMs completed the task). The LLMs (baseline) proposed 393 search terms, not necessarily including the original 20 from the clinicians’ original roadmap. While these search terms matched 12 151 ICD-10 codes, only 782 (6%) of those codes were observed in our sample.

### LLMs (context) roadmap

Second, building on the same function for the roadmap’s structure, the prompt was modified slightly to instruct the LLMs to include the examples from the clinicians’ original roadmap (Table 1) in each iteration (Prompt 2). Again, the *LLMs (context) roadmap* comprised all unique search terms across the 20 resulting dataframes.

Prompt 2: Generate LLMs (context) roadmap Please propose an exhaustive list of terms (avoiding acronyms) that will be used to search ICD-10 descriptions to identify each of the missing biomarkers. Create a new dataframe with these codes named “df_context_[number].” Be sure to include the examples given in (e.g.,) and make as exhaustive a list as possible.

Large language models (context) proposed 277 search terms, which matched 5661 distinct ICD-10 codes (514 in our sample, 9%). Notably, LLMs (context) proposed fewer search terms and had fewer matching ICD-10 codes than LLMs (baseline), but more of the suggestions were expected to be clinically relevant.

We previously ran both LLMs enhancements with prompts that (1) asked for “ICD codes,” but not ICD-10 specifically and (2) asked for all 20 dataframes at once, rather than in a loop. Based on these earlier versions, the LLMs proposed far fewer search terms. However, given that more than 6 months passed between then and the most recent results, it is unclear how much this change is attributable to prompting differences vs the LLMs’ improvements over time. See Supplementary Material S1 for more details.

### Clinicians’ adjudication on the LLMs (context) roadmap

We considered 1 final roadmap, where 2 clinicians independently reviewed the proposed LLMs (context) additions and adjudicated whether the search terms were valid for the corresponding ALI components. We focused on the LLMs (context), which was expected to outperform the LLMs (baseline).^72^ Of the 514 ICD-10 codes matching patients in our sample based on LLMs (context), at least 1 clinician endorsed 421 of them (82%).

Some disqualified terms were actually matches from the original clinicians’ roadmap, like “diabetes inspidus” or family histories of matching diseases. However, other exclusions, like “pulmonary hypertension” were new additions by the LLMs (context) that clinicians did not find clinically relevant for the corresponding ALI component. Still, the *LLMs (context + clinicians) roadmap* matched almost twice as many ICD-10 codes in our sample as the original.

## Results

### Descriptive statistics

The sample of N=1000 patients was 48 years old, on average, 40% male, 72% White, 18% Black, and 93% not Hispanic or Latino (Table 3). The subset of n=100 patients with chart reviews was slightly older (median age of 50 years) with higher percentages male (48%), White (74%), Black (20%), and not Hispanic or Latino (95%).

**Table 3:** Description of the original sample of N=1000 patients from the electronic health records at Atrium Health Wake Forest Baptist Hospital, further broken down by whether or not the patients underwent expert chart review.

		Expert chart review?	
	Overall	No	Yes
	(*N* = 1000)	(*N*−*n* = 900)	(*n*=100)
Patient demographics			
Age	48 (35, 57)	48 (35, 57)	50 (31, 59)
Sex			
Male	395 (39.5%)	347 (38.6%)	48 (48.0%)
Race			
American Indian or Alaska Native	6 (0.6%)	5 (0.6%)	1 (1.0%)
Asian Indian	30 (3.0%)	29 (3.2%)	1 (1.0%)
Black or African American	181 (18.1%)	161 (17.9%)	20 (20.0%)
Other	68 (6.8%)	64 (7.1%)	4 (4.0%)
White or Caucasian	715 (71.5%)	641 (71.2%)	74 (74.0%)
Ethnicity			
Hispanic, Latino or Spanish	61 (6.1%)	56 (6.2%)	5 (5.0%)
Not Hispanic, Latino or Spanish	934 (93.4%)	839 (93.2%)	95 (95.0%)
Patient refused	5 (0.5%)	5 (0.6%)	0 (0.0%)
Allostatic load index (ALI) and healthcare utilization			
Unvalidated ALI	0.33 (0.17, 0.50)	0.33 (0.17, 0.50)	0.38 (0.17, 0.57)
Missing ALI components	4 (3, 4)	4 (3, 4)	4 (3, 5)
Unique diagnosis codes	30 (16, 48)	30 (16, 48)	32 (17, 47)
Engaged in care	318 (31.8)	268 (29.8)	50 (50.0)

Categorical variables are summarized by their frequency (%). Numeric variables are summarized by their median (interquartile range). Unvalidated ALI and the number of missing components were taken from the extracted electronic health record data. Diagnosis codes were based on the ICD-10 (International Classification of Diseases, 10th revision). Patients with ≥1 emergency department visit or hospitalization were considered to have “engaged in care.”

According to extracted EHR data, the median ALI for patients undergoing chart review was slightly higher than that for the entire sample (0.38 vs 0.33). By design, a much higher proportion of patients undergoing chart review engaged in the healthcare system (50% vs 32%), meaning that they had at least 1 hospitalization or emergency department visit during the 2-year study period. (We intentionally sampled a 50/50 ratio of patients engaging/not engaging in the healthcare system for chart reviews.^26^) Patients were missing 4 ALI components, on average, whether or not they underwent chart review; however, those undergoing chart review had a slightly wider IQR of [3, 5] vs [3, 4]. On average, there were 30 unique ICD-10 codes per patient in the overall sample (IQR =[16,48]) but 32 each (IQR =[17,47]) among those with chart reviews.

We first compared the algorithm’s findings, based on various roadmaps, for the 100 patients who underwent chart reviews. We were interested in (1) how well the ALI components based on the algorithm agreed with those from the chart review and (2) why the algorithm might recover information that the chart review did not (and vice versa). Then, we scaled the algorithm up for use in the entire sample of 1000 patients.

### Expert chart reviews vs missing data recovery algorithms

A total of 1000 data points were considered in the chart reviews (10 ALI components for each of n=100 patients). Each data point was assigned to one of 4 mutually exclusive categories: unhealthy, healthy, missing, or “protocol error.” Data points classified as protocol errors were missing in the extracted EHR data, but, instead of following the roadmap, the chart reviewers found non-missing values that were outside the study period. These data points were returned to missing for our comparisons, while the algorithm might still recover them.

**Figure 1:**
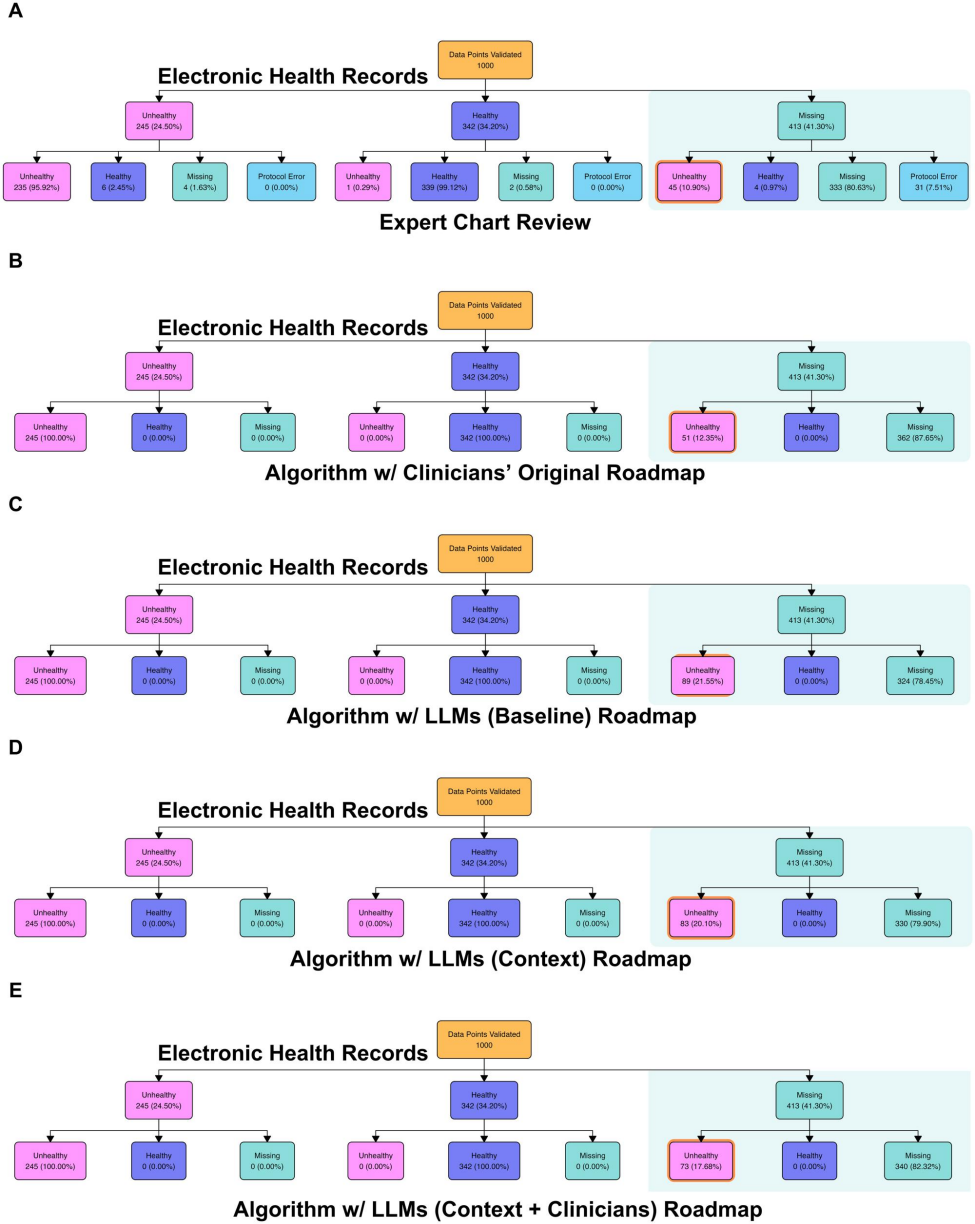
Flow charts of the 1000 data points included in the expert chart reviews (10 components per patient across n=100 patients), broken down by the status of the allostatic load index (ALI) components (unhealthy, healthy, or missing). We compare the extracted electronic health record (EHR) data to the expert chart reviews (subfigure A) and the missing data recovery algorithms based on the following roadmaps: clinicians’ original (subfigure B), large language models (LLMs) (baseline, subfigure C), LLMs (context, subfigure D), and LLMs (context + clinicians, subfigure E). Shaded boxes denote data points that were missing in the EHR data; these are the only data points that could potentially be changed by the missing data recovery algorithms in subfigures B-E, and a missing data point could only be changed to “unhealthy.”

Across all patients and components, 413 data points were missing in the extracted EHR data. The chart reviews recovered 45 (11%) that were presumed to be unhealthy (Figure 1A). (After accounting for protocol errors, this value differs slightly from our previous work^26^ but allows for a more fair comparison to the algorithms.) The amount recovered using the algorithm depended heavily on the roadmap (Figure 1B-E). The clinicians’ original recovered slightly more than the chart reviews (51, 12%). Large language models (baseline) recovered the most (89, 22%), followed by LLMs (context) (83, 20%). Large language models (context + clinicians) recovered more than the experts but less than the LLMs alone (73, 18%), suggesting that the enhancements successfully broadened the search terms but not all were clinically relevant.

**Figure 2:**
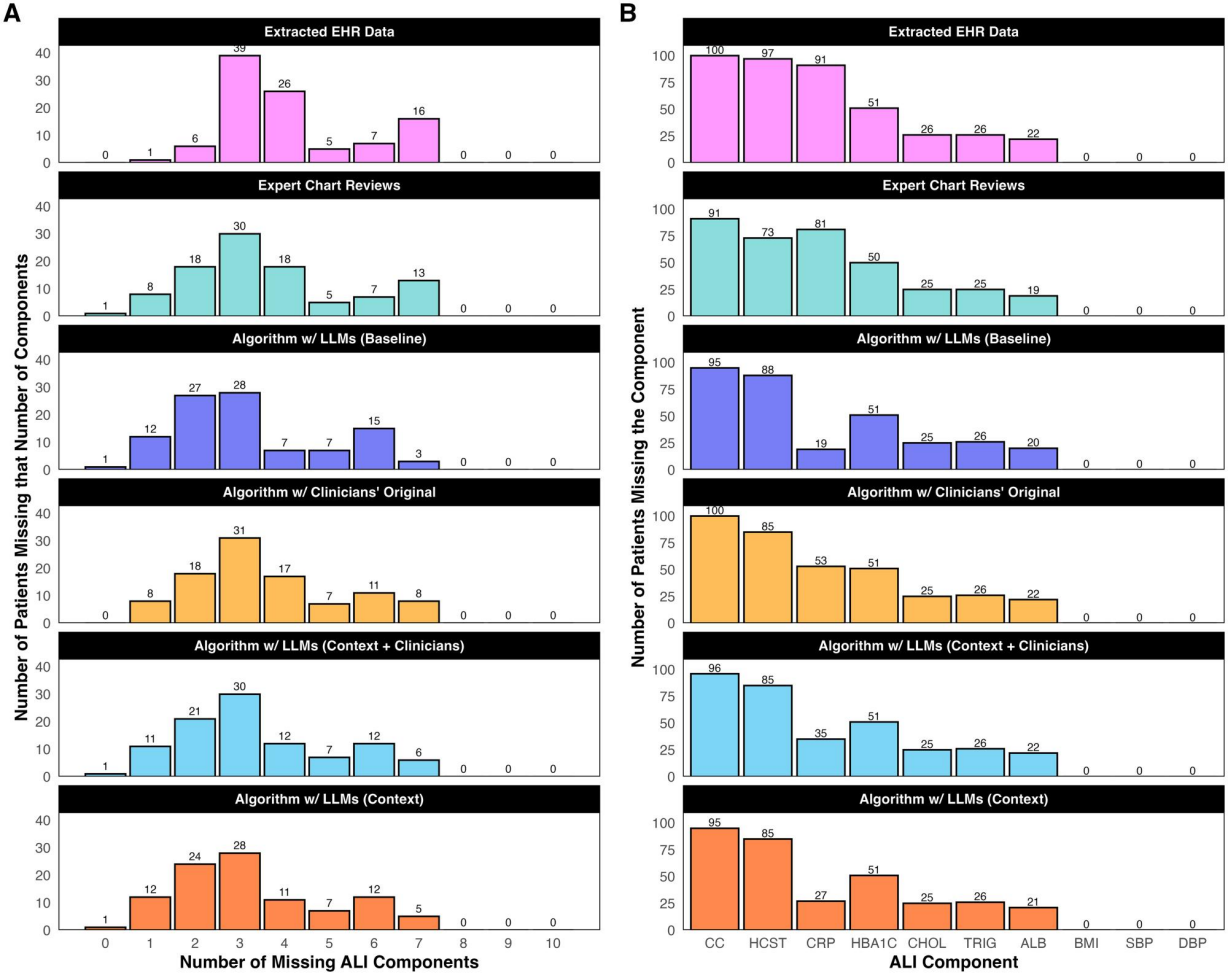
Bar plot of how many allostatic load index (ALI) components were missing per patient (subfigure A) and per component (subfigure B) across those chosen for expert chart reviews (n=100 patients). Missingness should be highest according to the unvalidated electronic health record (EHR) data, while the chart review might reduce it. The different missing recovery algorithms reduced the amount of missingness by varying amounts, depending on the roadmap: large language models (LLMs) (baseline), clinicians’ original, LLMs (context + clinicians), or LLMs (context). Acronyms for the ALI components: creatinine clearance (CC), homocysteine (HCST), C-reactive protein (CRP), hemoglobin A1c (HBA1C), total cholesterol (CHOL), triglycerides (TRIG), serum albumin (ALB), body mass index (BMI), systolic blood pressure (SBP), and diastolic blood pressure (DBP).

The counts of missing components per patient can be found in Figure 2A. In the extracted EHRs data, patients had 6 out of 10 non-missing ALI components, on average (IQR =[5,7]). Chart reviews improved this average to 7 out of 10 (IQR =[5.75,8]). All roadmaps led to the same improvement, but their IQRs differed slightly. The clinicians’ original roadmap had the widest IQR[5,8], while the LLMs (context + clinicians) roadmap led to the narrowest [6,8]. The other 2 roadmaps had the same IQR as the chart reviews.

The counts of missing values per component can be found in Figure 2B. No body mass index (BMI) or systolic/diastolic blood pressure values were missing. The chart reviews and algorithms all recovered 1 missing cholesterol component. Across the other 6 ALI components, 2 types of disagreement between the chart reviews and algorithms were possible.

First, the chart reviews could find something that the algorithm did not. Fortunately, there were relatively few of these. For hemoglobin A1c (HbA1c) and triglycerides, the clinical experts recovered 1 patient’s missing component that the algorithms did not. The chart reviewers recovered more patients’ homocysteine (24) and creatinine clearance (9), although these differences depended some on the roadmap. They likely had access to additional information in the electronic chart (e.g., lab values from previous providers). Similarly, no search terms were given for serum albumin, and yet the chart reviewers recovered 3 missing components.

Second, the algorithm could recover something that the chart reviews did not. We only observed this type of disagreement for CRP, where all roadmaps seemingly recovered more missing values (38-72 vs 10). These disagreements seemed to stem from clinical interpretation. “Infection” was a search term for CRP, and sometimes a matching diagnosis code was present in a patient’s chart but deemed not relevant by the chart reviewers (e.g., urinary tract infection). The LLMs (context + clinicians) roadmap excluded these diagnoses while building upon the original.

### Scaling up to the entire study

We applied the final algorithm, using the LLMs (context + clinicians) roadmap, to the entire 1000-person sample. Of 4009 missing ALI components, 789 (20%) were recovered. The median ALI was slightly higher after applying it (0.40 vs 0.33 in the extracted EHR data). The distribution of the ALI after recovery was also slightly more symmetric (Supplemental Figure S3) and less dispersed (IQR =[0.29,0.56] vs [0.17,0.50]).

**Figure 3:**
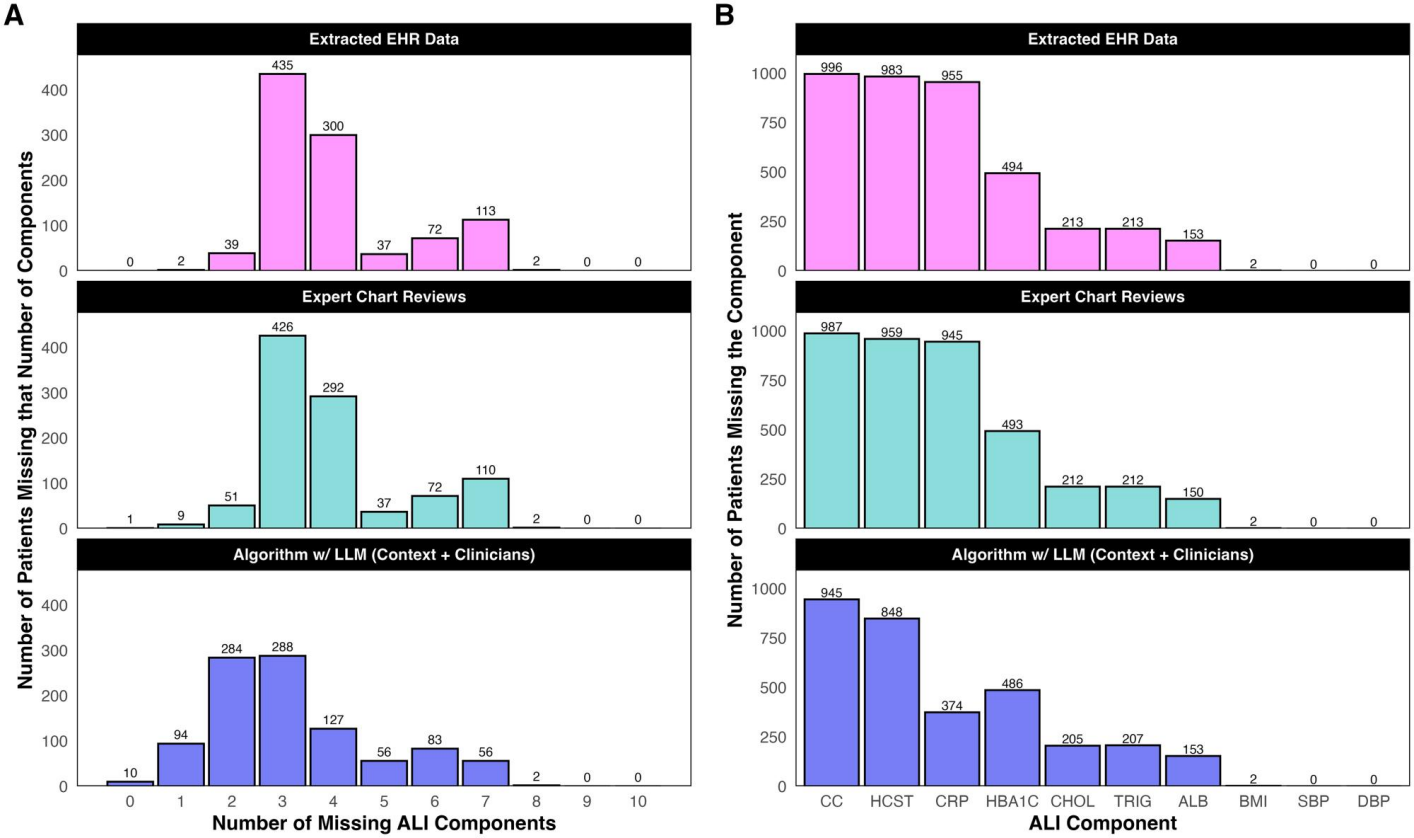
Bar plots of how many allostatic load index (ALI) components were missing per patient (A) and per component (B) across the full sample (N=1000 patients). Missingness should be highest according to the unvalidated electronic health record (EHR) data, while the chart review might reduce it. We only considered algorithmic recovery based on the large language models (LLMs) (context + clinicians) roadmap, which could be applied across the full sample and sometimes recovered more missing data than the expert chart reviews. Acronyms for the ALI components: creatinine clearance (CC), homocysteine (HCST), C-reactive protein (CRP), hemoglobin A1c (HBA1C), total cholesterol (CHOL), triglycerides (TRIG), serum albumin (ALB), body mass index (BMI), systolic blood pressure (SBP), and diastolic blood pressure (DBP).

The chart reviews did not move the sample-wide number of non-missing components per patient (median =6 with IQR =[6,7], as in the extracted EHR data), since they only helped 100 patients. Meanwhile, algorithmic recovery led to 7 non-missing ALI components per patient, on average (IQR =[6,8]), because the entire 1000-patient was eligible (Figure 3A).

The algorithm recovered missing values for 6 of 10 ALI components (Figure 3B). Noticeably, the most recovered components were for CRP (581 of 955, 61%), followed by homocysteine (135 of 983, 14%). Small percentages (≤5%) of missing creatinine clearance, HbA1c, cholesterol, and triglycerides components were also recovered. Again, no blood pressure values were missing. The only incomplete components that the algorithm did not help with were serum albumin (which only had a small number of clinician-approved terms recommended by the LLMs) and BMI (which was only missing for 2 patients).

**Figure 4:**
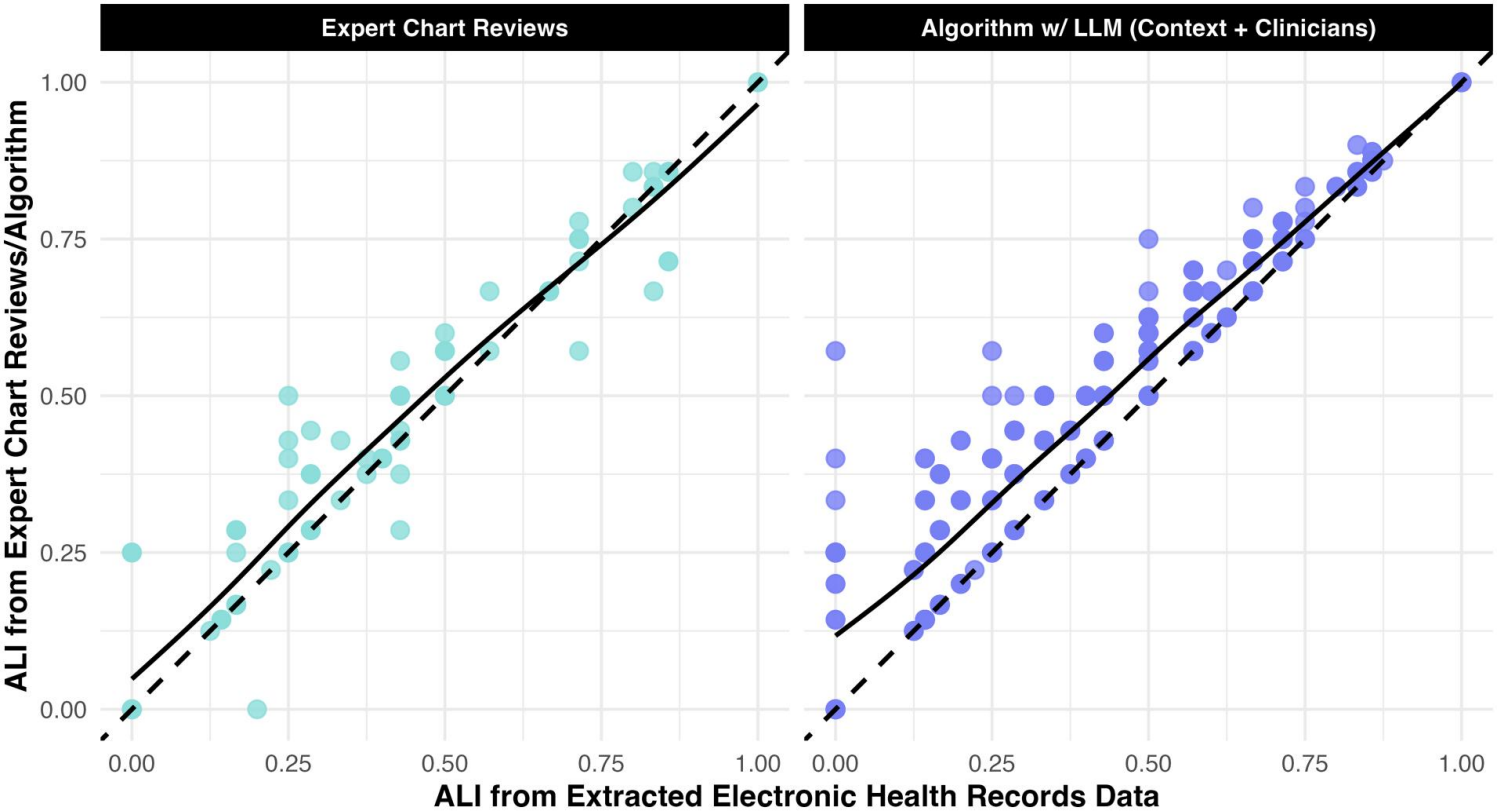
Many patients’ expert chart reviewed and algorithmically augmented allostatic load indices (ALIs) differed from the version in their extracted electronic health record (EHR) data. Still, the loess smoothers (solid lines) capturing the relationship between the validated/augmented and unvalidated ALIs were relatively linear and fell close to the line of equality (i.e., where unvalidated and validated/augmented ALI were equal).

The association between the ALI from the extracted EHR data vs the recovery algorithm was linear (Figure 4). This relationship, which could be captured with a statistical model, aligned closely with that between ALI from the expert chart reviews and extracted EHR data. If we wanted to attempt to predict a more accurate version of the ALI from EHR data, algorithmic recovery leads to similar values, on average, as having experts manually review a subset of patients.

**Figure 5:**
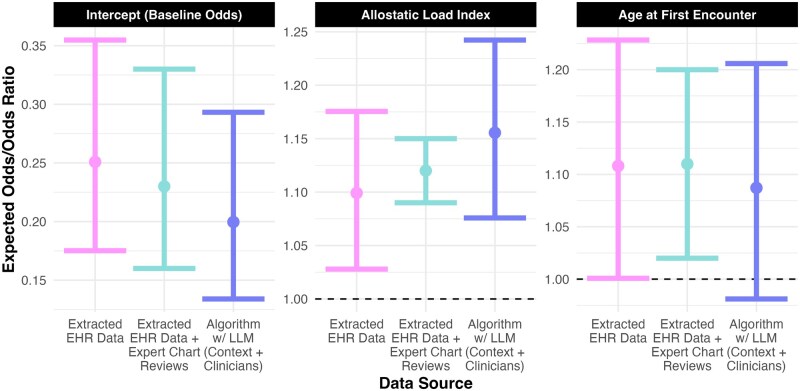
Coefficient estimates (with 95% confidence intervals) using the extracted electronic health record (EHR) data (naive analysis), combined EHR data + expert chart reviews (previous study estimates),^26^ and the missing data recovery algorithm using the large language models (LLMs) (context + clinicians) roadmap (augmented analysis).

Previously, we found ALI to be strongly associated with odds of engaging in care, after incorporating chart review and EHR data.^26^ (ALI from chart reviews was used in this model when available; otherwise, it was essentially predicted from the EHR data.) Here, we wanted to determine whether whole-sample algorithmic recovery could lead us to draw similar conclusions. Using logistic regression on the 1000 patients, we reestimated this model with only ALI from the extracted EHR data or algorithm. Overall, the algorithm-based analysis captured many of the same benefits as our incorporation of the chart reviews previously (Figure 5). Notably, our previous estimates had much better precision, because the study was specifically designed to do so by targeting the most informative patients for chart review.

## Discussion

Algorithmic recovery using clinically-driven roadmaps enhanced by LLMs could offer a cost-effective, scalable way to reduce missingness in EHR data before developing or validating computable phenotypes, like the ALI. Using the LLMs (context) expanded the search terms, while subsequent review by clinicians ensured that the final roadmap included only clinically-relevant ones (avoiding potential hallucinations). As proposed, the algorithm focuses on promoting completeness. Additional checks could be incorporated to address additional dimensions of EHR data quality, like correctness or plausibility.^66^ If using LLMs that run locally, such that no protected health information is shared, there are also exciting opportunities to enhance these quality checks using the LLMs on EHR data directly.^73^^,^^74^

We focused on operationalizing a well-studied, existing ALI calculation.^25^ Fortunately, our proposed algorithm generalizes easily to develop alternative ALI calculations or computable phenotypes for different outcomes. After identifying the variables needed and defining clinicians’ starting search terms, the algorithm would proceed as discussed.

Our roadmap-based logic is straightforward and efficient to incorporate into EHR software (e.g., as SQL code). Optimal presentation of the ALI in EHR is a key next step, as “alert fatigue” is a well-known source of burnout for clinical staff.^75^ Given the ALI’s simplicity and interpretability, there is an opportunity to present it to patients, perhaps through an EHR messaging portal.^76^ This connection could eventually become bi-directional, wherein patients upload data from personal devices (e.g., Oura ring)^77^ that can be incorporated in a future version of the ALI.

Our choice of general-purpose LLMs represents a practical decision and area for future methodological refinement. We chose to use Google Gemini’s free tier for accessibility and cost-effectiveness. However, there are many alternatives, including ChatGPT,^78^ Claude,^79^ Llama,^80^ and Copilot,^81^ each with capabilities that could influence search term generation quality. Our methodology only passed the roadmap structure to the LLMs, rather than any patient data, which eliminates privacy concerns and allows flexibility in model selection without regulatory constraints. Training LLMs specifically on ICD-10 codes and medical terminology could improve the validity and clinical relevance of proposed search terms. Future iterations would benefit from comparing performance across different LLMs providers and exploring domain-specific model training to optimize the balance between comprehensive coverage and clinical accuracy in missing data recovery algorithms.

There are many other worthwhile directions for future work. The expert chart reviews and algorithms pulled patients’ diagnosis codes at any time within the study period. If a relevant code was found, the ALI component was assumed to be unhealthy for the entire study. However, imposing a stricter lookback window (e.g., within 6 months of a visit with missing data) and exploring data-driven indications that the patient’s health may have improved (e.g., adherence to specific medications) could improve both approaches’ clinical relevance. Inviting patients to identify gaps in their own EHRs data (e.g., via survey) could provide valuable information about missingness mechanisms that would inform the roadmaps.^82–84^ Text-mining unstructured fields (like free-text notes) could uncover additional sources of auxiliary information.^85^

## Supplementary Material

ooag080_Supplementary_Data

## Data Availability

Due to patient privacy, the authors do not have permission to share the EHR data. Data for the roadmaps and algorithm are publicly available at https://github.com/lucymcgowan/ehr-llm-validation.
