# Supplementary material for: Using large language models to enhance clinically-driven missing data recovery algorithms in electronic health records
Source: JAMIA Open. 2026 Jun 2;9(3):ooag080. doi: 10.1093/jamiaopen/ooag080 (PMC13228140; doi:10.1093/jamiaopen/ooag080)
Supplement: ooag080_Supplementary_Data [file ooag080_supplementary_data.zip › Supp_ICD_Codes_EHR_Validation_Revision.pdf]

Supplemental Materials for "On Using Large Language Models to Enhance Clinically-Driven Missing Data Recovery Algorithms in Electronic Health Records"

Sarah C. Lotspeich, Abbey N. Collins, Brian J. Wells, Ashish K. Khanna, Joseph Rigdon, Lucy D'Agostino McGowan

R CODE

The following R code is also available for download at `https://github.com/LucyMcGowan/ehr-llm-validation/`.

Merge Roadmaps and ICD-10 Codes

We begin by reading in the ICD-10 codes with their diagnosis descriptions, which are archived on this GitHub. Then, we do minor string cleaning on the diagnosis descriptions (making them all capital letters and trimming potential whitespace) to make matching easier.

```
library(dplyr)
library(stringr)
library(tidyr)

# Read in ICD-10 codes with CDC descriptions (archived on GitHub)
icd10 = read.csv(file = "https://raw.githubusercontent.com/LucyMcGowan/ehr-llm-validation/refs/
                  heads/main/data-raw/icd10cm-codes-2026.csv") |>
  mutate(
    ## Convert descriptions to all CAPS for easier search
    DX_DESC = toupper(x = DX_DESC),
    ## Trim whitespace
    DX_DESC = str_trim(string = DX_DESC)
  )
```

Next, we read in a 10 x 2 dataframe for our chosen roadmap. Here, we demonstrate with the LLM (context) roadmap. After reading it in, we again clean the diagnosis descriptions.

```
# Read in LLM (context) roadmap
roadmap = read.csv(file = "https://raw.githubusercontent.com/LucyMcGowan/ehr-llm-validation/refs/
                    heads/main/data-raw/llm_context_roadmap.csv")

# Transform the roadmap into longer format
roadmap = roadmap |>
  ## Create separate rows for each variable, keyword combo
  separate_longer_delim(cols = If_Missing_Search_For,
                        delim = ";") |>
  mutate(
    ## Convert to all CAPS for easier search
    If_Missing_Search_For = toupper(x = If_Missing_Search_For),
    ## Trim whitespace
    If_Missing_Search_For = str_trim(string = If_Missing_Search_For),
    ## Remove punctuation
    If_Missing_Search_For = str_replace_all(string = If_Missing_Search_For,
                                            pattern = "[[:punct:]]",
                                            replacement = ""),
    ## Remove whitespce left behind
```

```
    If_Missing_Search_For = str_replace_all(string = If_Missing_Search_For,
                                            pattern =  "\\s+",
                                            replacement = ".*")
  )
```

Finally, we create a cross-join between the ICD-10 codes and the long version of the roadmap and check for matches.

```
# Create a crosswalk between ICD-10 codes and the roadmap (longer) and check for matches
matches = icd10 |>
  ## Consider all combinations of ICD-10 codes and roadmap search terms
  cross_join(roadmap) |>
  ## Subset to combinations where the ICD-10 code and search terms match
  filter(str_detect(string = DX_DESC,
                    pattern = regex(If_Missing_Search_For,
                                    ignore_case = TRUE)))
```

It is possible that multiple search terms match the same ICD-10 codes, so we then group them and summarize all matches per variable per diagnosis.

```
# Summarize all matching search terms per diagnosis/variable
matches = matches |>
  ## Group by variable and diagnosis
  group_by(Variable_Name, DX_CODE, DX_DESC) |>
  ## Combine all matching search terms, separated by semicolons
  summarise(matched_terms = str_trim(string = paste(If_Missing_Search_For,
                                                    collapse = "; ")),
            .groups = "drop")
```

Then, we merged the roadmaps into patients' ICD-10 codes extracted from the electronic health records (EHR).

Large Language Models (LLM) Enhancements

We implemented this *LLM-based roadmap enhancement* using the `ellmer` package in `R`, which enables programmatic interaction with LLM through "tool-calling."[1] That is, the `ellmer` package allows LLM to request execution of `R` functions as external tools. Specifically, we used Gemini-2.5-Flash[2] to test two LLM enhancements.

First, we provided the roadmap's structure as a dataframe "tool" that the LLM could access and modify when prompted. Specifically, we created a function that takes search terms for the ALI components (as vectors) and combines them following the clinical roadmap's structure (a 10×2 dataframe with a rows per component and a column for search terms). Next, we defined a tool that built on this function by adding a description of the tool's purpose,

> "Create a data frame with text ICD-10 description search terms separated by ; for ICD-10 descriptions to match diagnoses when missing from chart review,"

and arguments for each component of the roadmap framework, like

> "Search terms for ICD-10 text descriptions to detect diagnoses that would suggest that a patient's creatinine clearance is at an unhealthy (low) level, separated by a semicolon."

Then, through the `ellmer` package's tool functionality, we registered this tool (i.e., gave Gemini access to use it) and prompted the LLM to generate relevant terms for each ALI component. Essentially, we requested updates to this dataframe from the LLM, which `ellmer` executed within our `R` session based on either Prompt 1 or 2 from the main text. In this way, we obtained a programmatically-updated roadmap object that could be immediately used in our missing data recovery algorithm pipeline. The follow `R` code implements both of our LLM-based roadmap enhancements as described.

```
## Load package -----------------
library(ellmer)
library(dplyr)
```

```
## No context (no examples) -----
for (run in 1:20) {
  ### Initialize new chat -------
  c <- chat_google_gemini()

  ### Define make_data function -
  make_data <- function(creat_c, alb, bmi, sbp, dbp, a1c, chol, trig, crp, hcst, df_name) {
    df <- data.frame(
      Variable_Name = c(
        "CREAT_C",
        "ALB",
        "BMI",
        "BP_SYSTOLIC",
        "BP_DIASTOLIC",
        "A1C",
        "CHOL",
        "TRIG",
        "CRP",
        "HCST"
      ),
      If_Missing_Search_For = c(
        creat_c,
        alb,
        bmi,
        sbp,
        dbp,
        a1c,
        chol,
        trig,
        crp,
        hcst
      )
    )
    assign(df_name, df, envir = .GlobalEnv)
  }

  ### Define tool based on it ---
  tool_data <- tool(
    fun = make_data,
    description = "Create a data frame with text ICD-10 Description search terms separated by ;
                  for ICD-10 descriptions to match diagnoses when missing from chart review",
    arguments = list(
      creat_c = type_string(
        "Search terms for ICD-10 text descriptions to detect diagnoses that would suggest that a
        patient's creatinine clearance is at an unhealthy (low) level, separated by a semicolon."
      ),
      alb = type_string(
        "Search terms for ICD-10 text descriptions to detect diagnoses that would suggest that a
        patient's serum albumin is at an unhealthy (high) level, separated by a semicolon."
      ),
      bmi = type_string(
        "Search terms for ICD-10 text descriptions to detect diagnoses that would suggest that a
        patient's body mass index (BMI) is at an unhealthy (high) level, separated by a semicolon."
      ),
```

```
      sbp = type_string(
        "Search terms for ICD-10 text descriptions to detect diagnoses that would suggest that a
        patient's systolic blood pressure is at an unhealthy (high) level, separated by a semicolon."
      ),
      dbp = type_string(
        "Search terms for ICD-10 text descriptions to detect diagnoses that would suggest that a
        patient's diastolic blood pressure is at an unhealthy (high) level, separated by a semicolon."
      ),
      a1c = type_string(
        "Search terms for ICD-10 text descriptions to detect diagnoses that would suggest that a
        patient's hemoglobin A1c (HbA1c) is at an unhealthy (high) level, separated by a semicolon."
      ),
      chol = type_string(
        "Search terms for ICD-10 text descriptions to detect diagnoses that would suggest that a
        patient's total cholesterol is at an unhealthy (high) level, separated by a semicolon."
      ),
      trig = type_string(
        "Search terms for ICD-10 text descriptions to detect diagnoses that would suggest that a
        patient's trigylcerides is at an unhealthy (high) level, separated by a semicolon."
      ),
      crp = type_string(
        "Search terms for ICD-10 text descriptions to detect diagnoses that would suggest that a
        patient's C-reactive protein is at an unhealthy (high) level, separated by a semicolon."
      ),
      hcst = type_string(
        "Search terms for ICD-10 text descriptions to detect diagnoses that would suggest that a
        patient's homocysteine is at an unhealthy (high) level, separated by a semicolon."
      ),
      df_name = type_string("Name of the data frame")
    )
  )

  ### Register the tool -------
  c$register_tool(tool_data)

  ### Prompt chat -------------
  c$chat(
    paste0(
      "Please propose an exhaustive list of terms (avoiding acronyms) that will be used to search
      ICD-10 descriptions to identify each of the missing biomarkers. Create a new data frame with
      these codes named `df_nocontext_", run, "'. Be sure to make as exhaustive a list as possible.")
  )
}

## With context (examples) -----
for (run in 1:20) {
  ### Initialize new chat -------
  c_context <- chat_google_gemini()

  ### Define make_data function -
  make_data <- function(creat_c, alb, bmi, sbp, dbp, a1c, chol, trig, crp, hcst, df_name) {
    df <- data.frame(
      Variable_Name = c(
        "CREAT_C",
        "ALB",
```

```
      "BMI",
      "BP_SYSTOLIC",
      "BP_DIASTOLIC",
      "A1C",
      "CHOL",
      "TRIG",
      "CRP",
      "HCST"
    ),
    If_Missing_Search_For = c(
      creat_c,
      alb,
      bmi,
      sbp,
      dbp,
      a1c,
      chol,
      trig,
      crp,
      hcst
    )
  )
  assign(df_name, df, envir = .GlobalEnv)
}

### Add examples to tool_data -
tool_data <- tool(
  make_data,
  description = "Create a data frame with text ICD-10 Description search terms separated by ;
                for ICD-10 descriptions to match diagnoses when missing from chart review",
  arguments = list(
    creat_c = type_string(
      "Search terms for ICD-10 text descriptions to detect diagnoses that would suggest that a
      patient's creatinine clearance is at an unhealthy (low) level (e.g., renal failure, renal
      insufficiency, acute kidney injury, and chronic renal failure), separated by a semicolon."),
    alb = type_string(
      "Search terms for ICD-10 text descriptions to detect diagnoses that would suggest that a
      patient's serum albumin is at an unhealthy (high) level, separated by a semicolon."),
    bmi = type_string(
      "Search terms for ICD-10 text descriptions to detect diagnoses that would suggest that a
      patient's body mass index (BMI) is at an unhealthy (high) level (e.g., Obesity, morbid
      obesity, Grade I obesity, Grade II obesity, Grade III obesity), separated by a semicolon."),
    sbp = type_string(
      "Search terms for ICD-10 text descriptions to detect diagnoses that would suggest that a
      patient's systolic blood pressure is at an unhealthy (high) level (e.g., hypertension),
      separated by a semicolon."),
    dbp = type_string(
      "Search terms for ICD-10 text descriptions to detect diagnoses that would suggest that a
      patient's diastolic blood pressure is at an unhealthy (high) level (e.g., hypertension),
      separated by a semicolon."),
    a1c = type_string(
      "Search terms for ICD-10 text descriptions to detect diagnoses that would suggest that a
      patient's hemoglobin A1c (HbA1c) is at an unhealthy (high) level (e.g., diabetes, impaired
      glycemic control), separated by a semicolon."),
    chol = type_string(
```

```
      "Search terms for ICD-10 text descriptions to detect diagnoses that would suggest that a
      patient's total cholesterol is at an unhealthy (high) level (e.g., hypercholesterolemia),
      separated by a semicolon."),
    trig = type_string(
      "Search terms for ICD-10 text descriptions to detect diagnoses that would suggest that a
      patient's trigylcerides is at an unhealthy (high) level (e.g., hypertriglyceridemia),
      separated by a semicolon."),
    crp = type_string(
      "Search terms for ICD-10 text descriptions to detect diagnoses that would suggest that a
      patient's C-reactive protein is at an unhealthy (high) level (e.g., sepsis, infection,
      autoimmune inflammatory syndrome), separated by a semicolon."),
    hcst = type_string(
      "Search terms for ICD-10 text descriptions to detect diagnoses that would suggest that a
      patient's homocysteine is at an unhealthy (high) level (e.g., hyperhomocysteinemia,
      vitamin deficiency), separated by a semicolon."),
    df_name = type_string("Name of the data frame")
  )
)

### Register the tool -------
c_context$register_tool(tool_data)

### Prompt chat -------------
c_context$chat(
  paste0("Please propose an exhaustive list of terms (avoiding acronyms) that will be used to
  search ICD-10 descriptions to identify each of the missing biomarkers. Create a new dataframe
  with these codes named `df_context_", run, "`. Be sure to include the examples given in (e.g.,)
  and make as exhaustive a list as possible.")
)
}
```

PREVIOUS VERSIONS OF PROMPTS FOR LLMS ENHANCEMENTS

We previously ran both LLMs enhancements with prompts that (i) asked for ICD codes, but not ICD-10 specifically, and (ii) asked for all 20 dataframes at once, rather than in a loop. These alternative versions of Prompts 1 and 2, denoted by Prompts S1 and S2, respectively, follow. They were each called once to create the 20 separate dataframes, which were then used to create the superset for the roadmap. From there, everything else followed as in the main text.

Prompt S1: Generate LLM (baseline) roadmap

Please propose an exhaustive list of terms (avoiding acronyms) that will be used to search ICD descriptions to identify each of the missing biomarkers and create a dataframe with these codes. I want you to repeat this process 20 times, creating a new dataframe each time with each having a unique name starting with `df_nocontext`. Each time you repeat this, be sure to make as exhaustive a list as possible. These lists can vary.

Prompt S2: Generate LLM (context) roadmap

Please propose an exhaustive list of terms (avoiding acronyms) that will be used to search ICD descriptions to identify each of the missing biomarkers and create a dataframe with these codes. I want you to repeat this process 20 times, creating a new dataframe each time with each having a unique name starting with `df_context`. Each time you repeat this, be sure to include the examples given in (e.g.,) and make as exhaustive a list as possible. These lists can vary.

Based on these earlier versions of the prompts, both LLMs enhancements proposed far fewer search terms than in the main text. The older LLMs (baseline) proposed 656 search terms, not necessarily including the original 20 from the clinicians' original roadmap. While 539 of them matched potential ICD-10 codes, only 118 actually matched diagnoses for patients in our sample. The older LLMs (context) proposed 950 search terms, which matched 1853 distinct ICD-10 codes (275 in our sample). These values could be compared with those in Table 1 for more comparisons between the final LLMs enhancements versus these previous iterations.

ADDITIONAL FIGURES

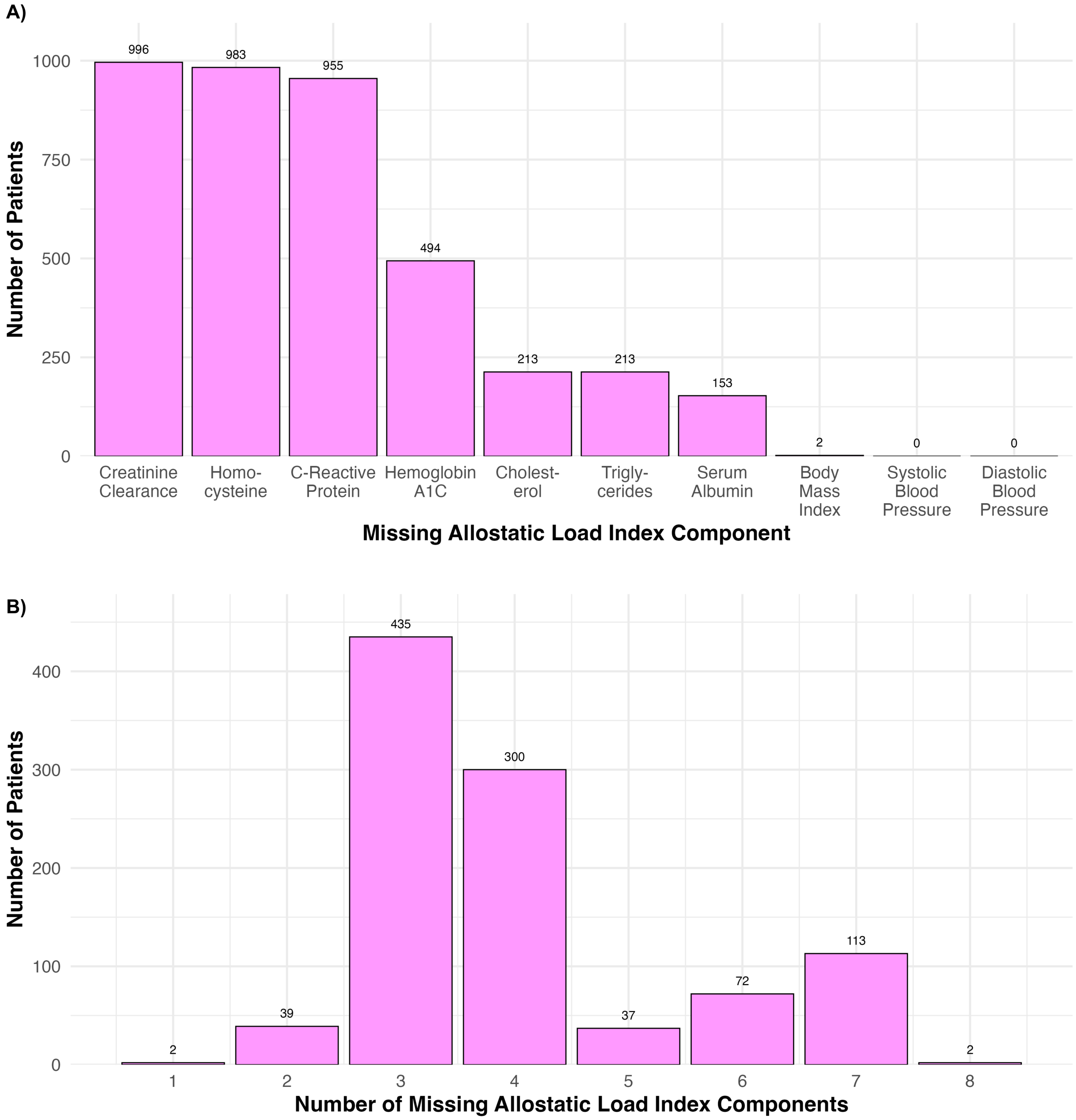

Figure S1. Counts of missing allostatic load index components **A)** per component and **B)** per patient from the original extracted electronic health records (EHR) data ($N = 1000$ patients).

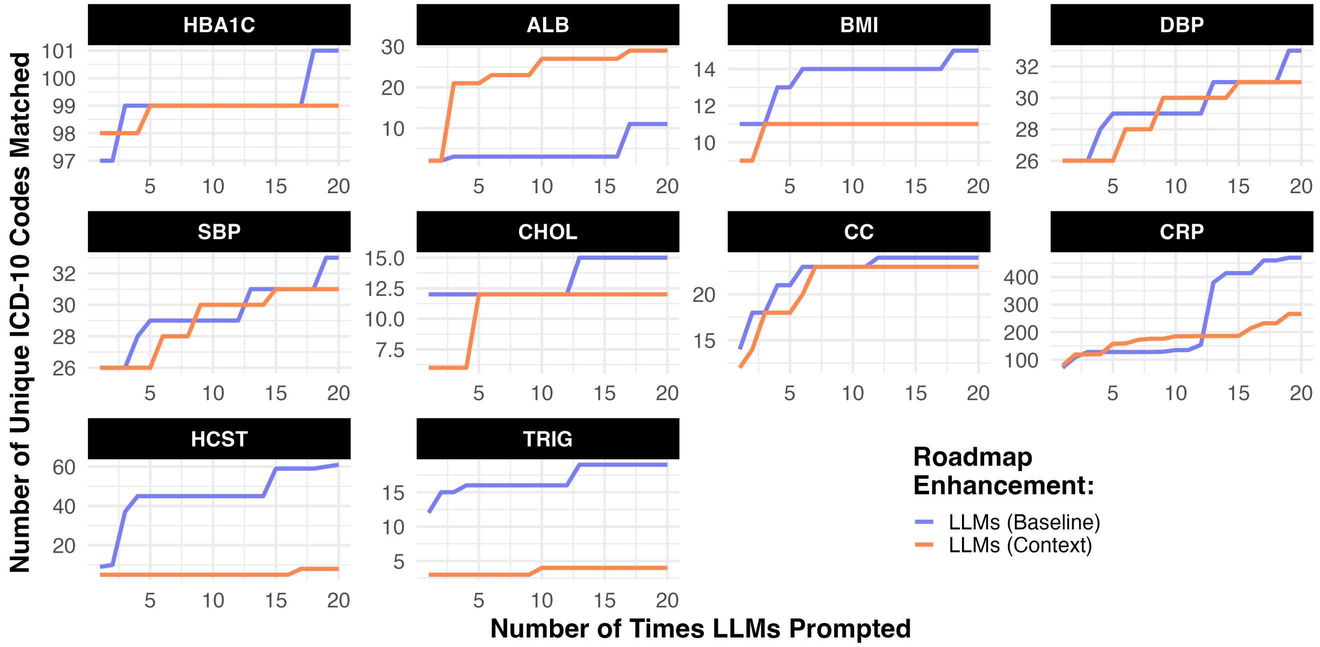

Figure S2. Counts of International Classification of Diseases (ICD-10) codes matching the superset of search terms proposed by the large language models (LLMs) as they were iteratively prompted 20 times with either Prompt 1 (Baseline) or 2 (Context). Acronyms for the allostatic load index (ALI) components: creatinine clearance (CC), homocysteine (HCST), C-reactive protein (CRP), hemoglobin A1c (HBA1C), total cholesterol (CHOL), triglycerides (TRIG), serum albumin (ALB), body mass index (BMI), systolic blood pressure (SBP), and diastolic blood pressure (DBP).

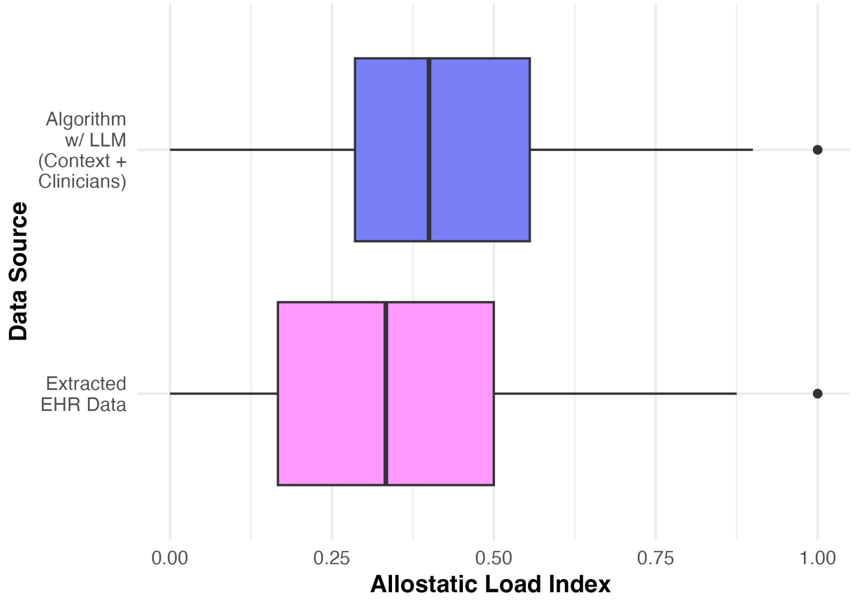

Figure S3. The median allostatic load index (ALI) for the 1000-patient study was slightly higher after applying the missing data recovery algorithm with the large language models (LLMs) (context + clinician) roadmap (0.40 versus 0.33 in the extracted EHR data). The distribution of the ALI after recovery was also slightly more symmetric and less variable (IQR $= [0.29, 0.56]$ versus $[0.17, 0.50]$).

REFERENCES

1. Wickham H, Cheng J, Jacobs A, Aden-Buie G, Schloerke B. ellmer: Chat with Large Language Models; 2025. R package version 0.3.0. Available from: `https://ellmer.tidyverse.org`.

2. Gemini Team Google. Gemini: A Family of Highly Capable Multimodal Models. arXiv preprint arXiv:231211805. 2023.
